# Is Mir-205 a possible biomarker for evaluating treatment response in psoriasis?

**DOI:** 10.25122/jml-2024-0264

**Published:** 2024-03

**Authors:** Carina Mihu, Codruța Alina Popescu, Diana Cenariu, Ştefan Vesa, Adrian Baican, Carmen Stanca Melincovici, Rareş Drulă, Adrian Bogdan Tigu, Anca Dana Buzoianu

**Affiliations:** 1 Department of Pharmacology, Toxicology and Clinical Pharmacology, Iuliu Hațieganu University of Medicine and Pharmacy, Cluj-Napoca, Romania; 2 Department of Human Sciences, Iuliu Hațieganu University of Medicine and Pharmacy, Cluj-Napoca, Romania; 3 Medfuture Research Centre for Advanced Medicine, Iuliu Hațieganu University of Medicine and Pharmacy, Cluj-Napoca, Romania; 4 Department of Dermatology, Iuliu Hațieganu University of Medicine and Pharmacy, Cluj-Napoca, Romania; 5 Department of Histology, Iuliu Hațieganu University of Medicine and Pharmacy, Cluj-Napoca, Romania

**Keywords:** mir-205, psoriasis, TNF-α

## Abstract

Psoriasis is a chronic skin disease that affects a significant number of patients and can severely impair quality of life. Although the diagnosis is normally clinical, paraclinical determination can occasionally be useful either in differential diagnosis or in evaluating the inflammatory response to treatment. MicroRNAs (miRNAs) are small non-coding parts of the RNA family that regulate gene expression and may have an important role as biomarkers in evaluating treatment response. The dysregulation of miRNAs has been well studied in other diseases, especially in oncology, but their role in chronic skin conditions such as psoriasis is still not fully understood. This study aims to evaluate the levels of three miRNAs (miR-155, miR-210, and miR-205) in patients with psoriasis, treated either systemically or topically, compared to a control group, and to assess the possible relationship between miRNA levels and systemic therapy. Our findings show a constant dysregulation of miR-205 in patients with psoriasis, with significantly higher levels compared to the control group, which can be explained as conferring a protective effect to treated patients. Further studies are needed in order to fully understand the role of miRNAs in the physiopathology of psoriasis and even, potentially, to provide more targeted genetic therapies in the future.

## INTRODUCTION

Psoriasis is an inflammatory disease affecting around 125 million people across the world, with a prevalence varying from 0.33% to 0.6% [1,2]. The recurrent chronic evolution of skin lesions has been explained by a more accentuated and rapid turnover of epidermal cells, leading to incomplete scaly lesions. Although it may appear simple on initial inspection, the physiopathology of this inflammatory disease is far more complex. It is very important to understand and continually study the inflammatory pathways involved to enhance the effectiveness of existing treatments, which have already improved quality of life for patients with this disease.

MicroRNAs represent small non-coding parts of the RNA family that are thought to have crucial roles in both physiological and pathological processes by regulating gene expression [3]. There are numerous studies that have proven the significant involvement of miRNAs in the pathogenesis of psoriasis [4–6]. It is well known that miRNAs have essential roles in influencing a cell’s life from growth to apoptosis, as well as its morphological and structural development. Despite these well-established characteristics, less is known regarding their impact on chronic inflammatory skin conditions [7]. Given that psoriasis is one of the most frequent and common inflammatory skin diseases with a high tendency to chronicity, it would be interesting to know more about the role that certain miRNAs might have in this condition, and even to start developing new gene therapies [8].

The pathological pathway starts with a large number of CD4+ and CD8+ T cells being present in the epidermis and upper dermis, which leads to the production of inflammation mediators such as tumor necrosis factor α (TNF-α), interleukin (IL)-2, IL-17, and IL-6 [9,10]. To fully understand the mechanisms involved and develop targeted treatments, we need to more closely examine the regulatory factors that lead to this proinflammatory cycle. An important differentiating factor in the T helper (Th) 2 cells is transcription factor GATA3. It has been proven that there is a downregulation of GATA3 in psoriasis lesions, possibly leading to the final part of the proinflammatory pathway, an increased turnover of epidermal cells and, therefore, clinical implications. A significant role in the inflammation pathway is played by gamma delta T cells with significant involvement in the proliferation of keratinocytes and, thus, in the inflammatory cycle. Although not as well studied as alpha beta T cells, the literature has identified their major role in innate immune response during inflammation and cytokine production, leading to the chronicity of this skin disease. The role of gamma delta T cells has crucial implications for modern targeted therapies owing to their role in the production of IL-17 [11,12]. More than 250 microRNAs (miRNAs) have been studied related to the pathogenesis of psoriasis, being detectable both in the lesional skin and the peripheral serum. Studies have shown that miR-155, a single-stranded, non-coding, short RNA molecule, also plays a role in the genetics behind this disease by suppressing the expression of GATA3 and, finally, modulating the production of one of the most important inflammatory factors, IL-6 [13]. Another important miRNA in the evolution of psoriatic lesions is miR-205-5p, which is considered to have a protective role in the physio-pathological path of this disease [14]. Finally, miR-210 has shown overexpression in the serum of patients with psoriasis, further leading to an increase in the Th1/Th17 axis and thereby having a vital role in the inflammatory cycle [15,16].

### MiR-205

Various roles for miR-205 have been described in the literature, being involved not only in physiological processes, such as tissue homeostasis, but also in the pathophysiology of neoplasia, such as bladder, prostate, and breast cancers. Because of its important dysregulations, miR-205 has been used as a biomarker in the diagnosis and staging of several cancers, but most importantly, it has proven to be a reliable marker in evaluating treatment response [17]. The most important role of miR-205 described in oncogenesis is its capacity to inhibit proliferation, being a positive prognosis marker of treatment response [18]. Regarding its involvement in skin pathologies, miR-205 has shown a high expression in the epidermis, having an important role in epithelial biogenesis [17].

### MiR-155

MiR-155 is molecule thought to be involved in a wide variety of immune-related processes that lead to abnormal skin cell proliferation and chronic inflammation. One of the most important roles that miR-155 reportedly has in the pathogenesis of psoriasis is the regulation of T cells and dendritic cells, but it also modulates inflammatory cytokines such as IL-17, IL-23, and TNF-α. MiR-155 can also influence keratinocyte proliferation, having an important role in the clinical manifestation of this skin condition [19]. Luo *et al*. discovered increased levels of miR-155 in psoriasis-affected skin and concluded that miR-155 most likely acted as a promoter in psoriasis by modifying gene expression [20].

### MiR-210

The role of miR-210 in psoriasis is less studied than the previously presented miRNAs, but it is thought that hypoxia, which is possibly involved in the pathogenesis of psoriasis, can induce the production of this miRNA. Another possible role is abnormal angiogenesis under hypoxic conditions in the psoriatic skin. All three of these miRNAs, including miR-210, are responsible for hypoxia-induced chronic inflammation and abnormal keratinocyte proliferation [4,21,22].

In this study, we aimed to investigate whether patients with psoriasis have higher serum levels of miR-155 that lead to the downregulation of serum levels of IL-6. Moreover, we aimed to determine whether miR-205-5p levels are downregulated or upregulated, considering its protective effect on psoriatic lesions and, more importantly, considering that our study focuses on both treated and non-treated patients [12]. The same approach was applied to miR-210, which was expected to have higher levels in patients with psoriasis, but given that our patients were already undergoing different types of treatments, we expected to find downregulation of this miRNA. We also aimed to assess the correlation between the serum level of miR-210-5p and that of the most important proinflammatory factor, TNF-α. The originality of this study lies in the assessment of miR-155, miR-210, miR-205, and TNF-α in already-treated patients with psoriasis.

## MATERIALS AND METHODS

### Data collection

The study included 32 patients with psoriasis who were either treated or not yet treated, and 31 patients without psoriasis or other inflammatory autoimmune diseases. These patients were recruited between July 2021 and November 2022 from patients presenting either for a consultation or for hospitalization to the Dermatology Department of Cluj County Emergency Hospital.

### Inclusion and exclusion criteria

We included adult patients above 18 years with any form of psoriasis (vulgar or plaque psoriasis, guttate psoriasis, scalp psoriasis, inverse psoriasis, palmoplantar psoriasis, pustular psoriasis, and nail psoriasis), with or without systemic treatment, and with or without active lesions at the time of the investigation. In terms of ongoing treatment for psoriasis, we included either non-treated patients or patients treated for more than 2 years with systemic treatments to determine whether treatment could influence not only the inflammatory cytokine profile leading to clinical amelioration but also the non-coding parts of the RNA.

We excluded patients with other autoimmune and inflammatory skin or articular diseases, such as pemphigus, bullous pemphigoid, scleroderma, lupus, rheumatoid arthritis, and ankylosing spondylitis, and intestinal autoimmune diseases such as Crohn’s disease and ulcerative colitis. We also excluded patients treated with systemic immunosuppressives or biotherapies for any other diseases except for psoriasis.

### Indicators

Peripheral blood samples were obtained from both patients and controls for the purpose of assessing the levels of variables, including pro-inflammatory cytokines (TNF-α, Il-6, and Il-12) and the non-coding parts of the RNA (miR-155, miR-205, and miR-210).

### Study population

Of the total number of patients diagnosed with psoriasis, we included 11 patients without any systemic treatment, treated only with local treatments such as dermocorticoids, calcipotriol, and emollients, none of which should influence our genetic determinations; 3 patients were systemically immunosuppressed with methotrexate, and 18 patients received biotherapies such as anti-TNF-α (adalimumab), anti-IL-17A (ixekizumab), and anti-IL-23 (secukizumab). Furthermore, 18 patients had no cardiovascular comorbidities, and 14 were suffering from various cardiovascular diseases such as arterial hypertension and ischemic heart disease.

### Determination methods

#### Plasma separation and peripheral blood mononuclear cell isolation

Non-fasting blood was collected in EDTA vacutainer tubes, and plasma was separated within 2 h and stored at −80 °C. The cell fraction was further used for PBMC isolation using Ficoll-Paque (GE Healthcare). The blood samples were diluted 1:1 with PBS and gently pipetted on top of the Ficoll solution in a 15 ml Falcon tube. The separation was performed at 20 °C using a swing-out rotor, at 620g, for 30 min with slow acceleration and gentle breaking. The buffy coat was then aspirated and washed with cold PBS, and the washing step was repeated three times at 1,000g for 15 min at 4 °C. The cell pellet was stored in 800 µl of Trizol and immediately frozen in liquid nitrogen. The plasma samples were further used for ELISA analysis, and the Trizol samples were used for RNA extraction and isolation, and miRNA analysis.

#### Immunoenzymatic testing (ELISA)

The plasmatic levels of IL-6, IL-12, and TNF-α were quantitatively evaluated with the ELISA immunoenzymatic method, following the manufacturer’s protocol for each ELISA assay kit: human IL-6 ELISA kit (cat no. E-EL-H6156), human IL-12 ELISA lit (cat. no. E-EL-H0150) and human TNF-α ELISA kit (cat no. E-EL-H0109), all purchased from Elabscience. The plates were read at 450 nm using a Tecan Spark 10M spectrophotometer (Tecan Group).

### RNA extraction

Total RNA was extracted from the PBMCs using the TripleXtractor reagent (GeneAll Biotechnology) in accordance with the manufacturer’s instructions. This process involved cell lysis in TripleXtractor, phase separation by chloroform, RNA precipitation with isopropanol and RNA pellet washing in 75% ethanol, followed by dissolving in RNase-free water. The RNA concentration and purity were evaluated using a NanoDrop spectrophotometer (Thermo Fisher Scientific).

### MiRNA quantification

Quantification of miR-155-5p, miR-205-5p, and miR-210-5p levels was performed using TaqMan Advanced miRNA Assays (Applied Biosystems). For each assay, 10 ng of total RNA from the samples was reverse-transcribed into cDNA, which was then diluted 1:5 in nuclease-free water. qPCR was performed using SsoAdvanced Universal Probes Supermix (Bio-Rad) in a 10 µl reaction volume per well in 96-well plates, on a StepOnePlus Real-Time PCR System (Applied Biosystems), with the following cycling conditions: an initial denaturation at 95 °C for 10 min, followed by 40 cycles of 95 °C for 15 s and 60 °C for 1 min. The relative expression of these miRNAs was calculated using the 2^-ΔΔCt method, which was normalized against miR-16-5p as the endogenous control.

### Statistical analysis

Statistical analysis was performed using the GraphPad Prism 8 software (GraphPad), using the unpaired *t*-test with Welch’s correction. Statistical significance was reported as a *P* value of ^*^<0.05, ^**^<0.01 and ^***^<0.001. Medical statistical analysis was carried out using MedCalc Statistical Software v.22.021 (MedCalc Software). To accurately represent the non-normally distributed data (tested with the Shapiro–Wilk test), quantitative variables were summarized as median and interquartile range (25th–75th percentiles), whereas qualitative variables were described using frequency and percentage distributions. Comparisons between groups were carried out using the Mann–Whitney test or Kruskal–Wallis test, whenever appropriate. A significance level of *P* < 0.05 was adopted to determine statistical significance.

## RESULTS

Most patients were diagnosed with vulgar psoriasis, the most common form of psoriasis, fewer than five patients being diagnosed with other forms. Although histopathological confirmation is not mandatory according to the protocol, 16 patients were also biopsied at the initial diagnosis. To evaluate the histopathological severity of the disease, we graded the findings according to the histological grading system for psoriasis vulgaris [23]. This system scores the following histopathological findings: regular elongation of the rete ridge, 1 point; club-shaped rete ridges, 2 points; elongation and oedema of the dermal papillae, 1 point; perivascular mononuclear infiltrate in the upper dermis of papillae, 1 point; absent granular layer, focal 1 point; absent granular layer, total 2 points; focal parakeratosis, 1 point; total parakeratosis, 2 points; suprapapillary plate thinning, 2 points; mitosis above basal cell layer, 2 points; Munro micro-abscesses, 3 points; and spongiform pustule, 3 points. The maximum score is 19. Out of the 16 biopsied patients, four patients had a score under 5, nine patients had a score between 5 and 10, and 13 patients had a score higher than 10. The maximum score was 11.

We compared the levels of the three inflammatory cytokines using Mann–Whitney *U* and Wilcoxon *W* tests in the control and psoriasis groups.

### Pro-inflammatory cytokines

**Figure 1:**
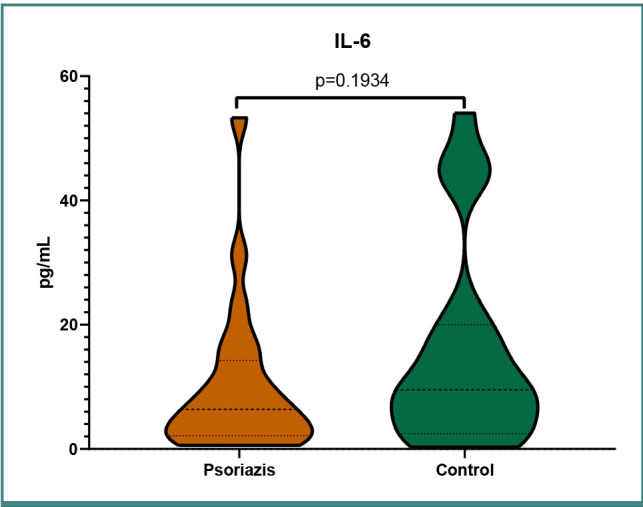
IL-6 levels in the psoriasis group and control group

**Figure 2:**
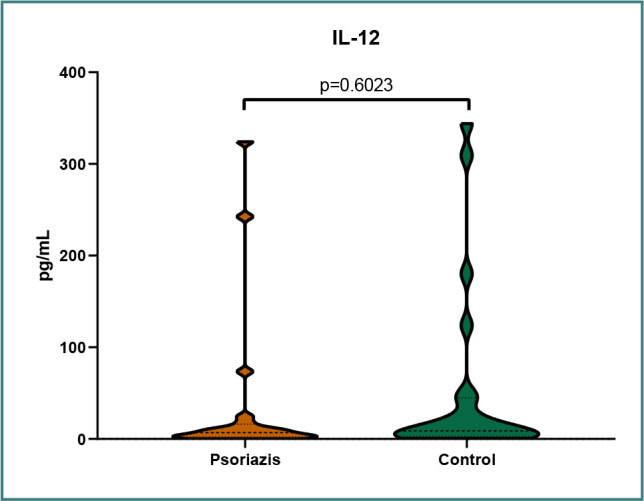
IL-12 levels in the psoriasis group and control group

**Figure 3:**
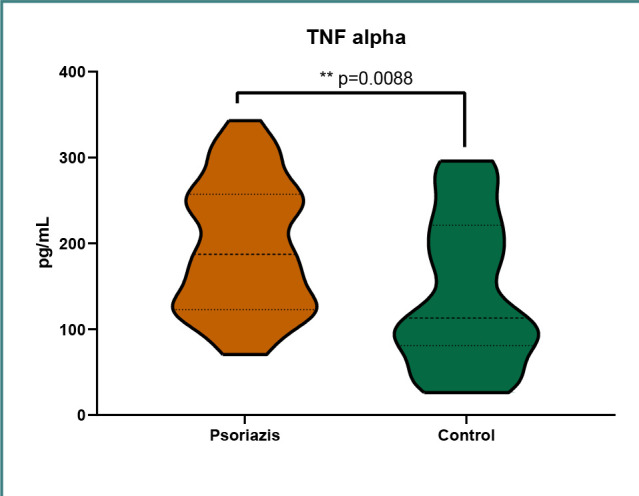
TNF-α levels in the psoriasis group and control group

The distribution of IL-6 levels in the two groups is presented in Figure 1. The distribution of IL-12 levels in the two groups is presented in Figure 2. The distribution of TNF-α levels in the two groups is presented in Figure 3.

We obtained statistical significance with a *P* value of <0.05 only for TNF-α, with significantly higher levels in the psoriasis group. For the other two inflammatory cytokines, IL-6 and IL-12, we were unable to obtain statistically significant data, most likely owing to the small group of patients, although the data suggested lower levels of both IL-6 and IL-12 in the psoriasis group. Based on the pathophysiology of the disease, we expected to find lower IL-6 and IL-12 levels in treated patients with psoriasis. The lower IL-12 levels we found probably indicate a positive response to treatment; however, IL-6 levels remained constant, being lower than in the control group. Moreover, most of our patients were treated with biological therapies targeting IL-17 and IL-23. These treatments did not influence TNF-α levels, which remained higher compared to the control group (Table 1).

**Table 1:** Comparison of inflammatory cytokine levels (TNFα, IL-6, and IL-12) between the psoriasis and control groups

	Control	Psoriasis	*P* value
TNF-α	113 (80.82–221.23)	187.20 (122.71–257.25)	0.007^*^
IL-6	7.26 (0–15.93)	2.61 (0–9.84)	0.440
IL-12	3.15 (0–20.05)	1.92 (0–9.61)	0.341

* Statistically significant. Data expressed as mean (range).

Only 15.6% of the patients in the psoriasis group received systemic treatment targeting TNF-α. Naturally, the anti-TNF-α subgroup had the lowest levels of TNF-α, but these levels were still higher than those in the control group. These findings suggest that more targeted treatments, such as anti-IL-17 or anti-IL-23, have no influence on TNF-α. On the other hand, we can see a normalizing trend for IL-6 and IL-12. As these two interleukins are not directly targeted by psoriasis treatment, we could not establish any statistical difference between the psoriasis and control groups. Table 2 presents a comparison of inflammatory cytokines within the psoriasis group, categorized by systemic treatment.

**Table 2:** Comparison of inflammatory cytokine levels in the psoriasis group based on the type of systemic treatment

	No treatment	Anti-TNF-α	Anti-IL-17/23	*P* value
**TNF-α**	175 (120.89–256.79)	168.84 (115.98–271.82)	221.71 (135.62–282.64)	0.598
**IL-6**	5.34 (0–17.87)	2.16 (0.41–8.47)	0.82 (0–5.87)	0.236
**IL-12**	0.05 (0–8)	11.52 (5.57–283.47)	1.68 (0–5.07)	0.101

Data expressed as mean (range).

No statistical significance was found when comparing the levels of the three inflammatory cytokines based on the type of treatment (no treatment, anti-TNF-α, and anti-IL-17/23). On the other hand, we found the lowest levels of TNF-α in patients undergoing anti-TNF-α therapies, which was expected. For the other two interleukins, larger groups of patients must be recruited in future studies in order to be able to describe patterns related to the treatment being taken.

### miRNA analysis

**Figure 4:**
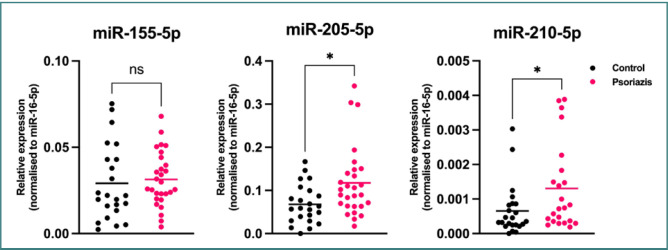
MiR-155, miR-205, and miR-205 levels in the psoriasis group and control group

The distribution of miR-155, miR-205, and miR-210 levels in the two groups is presented in Figure 4 and Table 3. The comparison of miRNA levels revealed that miR-205 levels were significantly higher in the psoriasis group (*P* = 0.031).

**Table 3:** Comparison of miRNA levels between the psoriasis and control groups

	Control	Psoriasis	*P* value
**miR-155**	0.0220 (0.1405–0.0429)	0.0304 (0.0233–0.0448)	0.130
**miR-205**	0.0612 (0.0323–0.1223)	0.1060 (0.0635–0.1544)	0.031^*^
**miR-210**	0.0004 (0.0002–0.0008)	0.0006 (0.0002–0.0015)	0.347

* Statistically significant. Data expressed as mean (range).

Our next aim was determine whether response to treatment can also affect miRNA levels in addition to inflammatory cytokine levels. The comparative analysis of miR-155, miR-205, and miR-210 levels in the psoriasis group, categorized by systemic treatment, is presented in Table 4. We did not find a statistically significant difference between miRNA levels according to the type of treatment used. The lowest levels of miR-155 and miR-205 were found in patients receiving no treatment.

**Table 4:** Comparison of miRNA levels within the psoriasis group, categorized by systemic treatment

	No treatment	Anti-TNF-α	Anti-IL-17/23	*P* value
**miR-155**	0.0255 (0.0225–0.0370)	0.0494 (0.0315–0.7625)	0.0320 (0.0239–0.0441)	0.103
**miR-205**	0.1021 (0.0627–0.1661)	0.2304 (0.1123–10525.28)	0.0895 (0.0632–0.1189)	0.130
**miR-210**	0.0005 (0.0002–0.0014)	0.0035 (0.0008–0.7509)	0.0003 (0.0002–0.0013)	0.319

Data expressed as mean (range).

## DISCUSSION

### Upregulation of miR-205

The major contribution of this study is that we found a statistically significant difference in miR-205 levels between already-treated patients with psoriasis and the control group, suggesting an upregulation of miR-205 in patients with psoriasis. We interpreted this as a protective phenomenon for our patient group due to the treatment that led to a clinically silent disease, a hypothesis also confirmed by Xue *et al*. [14]. To our knowledge, there are no studies in the literature on the influence of psoriasis treatments on the dysregulation of miR-205. Our study could be a first step toward determining the influence of modern targeted treatments on the genetic pattern of psoriasis to better understand the potential of newer treatments. Biological treatments used for psoriasis, such as TNF-α inhibitors (e.g., etanercept, adalimumab, or infliximab), IL-17 inhibitors (e.g., secukinumab or ixekizumab), and IL-23 inhibitors (e.g., ustekinumab or guselkumab), have been shown to modulate various aspects of the immune response’s involvement in the pathogenesis of psoriasis [24]. However, the specific effects of these treatments on miR-205 levels in patients with psoriasis have not been extensively studied.

The literature presents contradictory findings regarding the dysregulation of miR-205 in non-treated patients with psoriasis. Xue *et al*. have shown that the artificial upregulation of miR-205 could have a protective effect on patients with psoriasis [14], which supports our finding of the upregulation of miR-205 in treated patients. On the other hand, in a small study involving 13 patients with psoriasis and 13 controls, Zilbert *et al*. examined several miRNAs, including miR-205 from affected skin, by performing skin biopsies. They found that miR-205 could have an important role in psoriatic lesions by targeting SHIP2, a regulator of Akt signaling and phosphorylation of Bcl-2-associated death promoter (BAD), and by inhibiting apoptosis [25]. The anti-apoptotic role of miR-205 could indicate a negative effect on psoriasis. By contrast, the study of Xue *et al*. demonstrated downregulation in skin affected by psoriasis [14,25]. Because of the controversial findings reported in these pilot studies, further research is needed to fully understand the molecular mechanisms underlying the involvement of miR-205 in psoriasis and, potentially, its therapeutic target.

### Upregulation of TNF-α

Regarding the most important inflammatory cytokine, TNF-α, we were able to demonstrate a highly significant difference in TNF-α levels between the psoriasis and control groups. This shows a constant silent inflammation and also the most probable reason behind the chronicity of this disease. Therefore, regardless of the treatment used, TNF-α levels were constantly significantly higher in the psoriasis group, making it possible to rebound at any time, especially when trying to end the treatment. Interestingly, we noticed higher TNF-α levels even in the subgroup treated with anti-TNF-α therapies (adalimumab) compared to the control group. Although TNF-α is a well-studied molecule with numerous proven cases of involvement in psoriasis [26,27], we consider our findings important as the constant high levels of TNF-α despite systemic treatment could be a straightforward explanation for the chronicity of psoriasis.

### A possible role of miR-205 in downregulating TNF-α

We assumed that there is potentially a direct correlation between miR-205 regulation and TNF-α, in which the upregulation of miR-205 will consecutively determine downregulation of TNF-α and, thus, a reduction in chronic inflammation and improvement of skin lesions. Our assumption was made based on a study on the expression of miR-205 and other inflammation factors in chronic periodontitis [28], as we were not able to find studies referring strictly to the correlation between miR-205 and TNF-α in psoriasis. Although not referring to our studied pathology, we assumed that the chronic inflammation in these two different pathologies could have similar implications. Our results show upregulation of miR-205 and higher TNF-α levels in patients with psoriasis than in controls. Specifically, TNF-α levels were lower in patients undergoing anti-TNF-α treatments compared to non-treated patients and patients treated with other systemic treatments, which was expected. Most importantly, the highest miR-205 levels were found in patients undergoing anti-TNF-α therapies. These findings confirm our hypothesis that upregulation of miR-205 is correlated with lower TNF-α levels in patients with psoriasis. To our knowledge, there are no other studies in the literature that describe our hypothesis regarding patients with psoriasis undergoing treatment.

### The implications of treatment for miRNA levels

We tried to elucidate whether the dysregulation of the three miRNAs we studied depended on the systemic treatment received by the patients in the psoriasis group. Although our findings were not statistically significant, most probably because of the small number of patients, we found significant downregulation of miR-205 in the group treated with anti-IL-17 or anti-IL-23 therapies, compared to the other two groups with no treatment and anti-TNF-α treatments. Similar findings were obtained for the other two miRNAs, miR-210 and miR-155. The lowest levels of the three miRNAs were found in the anti-IL-17/anti-IL-23 group, followed by the no-treatment group and then the anti-TNF-α group. Further studies should be conducted to confirm our hypothesis that some systemic treatments could influence the genetic background of the disease.

### Study limitations

This study was a transversal pilot study conducted on a small group of 63 patients. We only obtained statistically significant data for TNF-α and miR-205, but the other statistical data suggest possible significance if tested in larger groups. Therefore, we consider the size of our study group and the heterogeneity in systemic treatment to be the most important limitations. Another possible limitation to be taken into consideration is the method used for genetic determination, using plasma from peripheral blood, whereas most of the other studies used skin samples from psoriasis-affected skin [29,30]. The reason we chose this method for our study was because our hypothesis was designed to determine whether inflammatory cytokines and non-coding parts of RNA are influenced by systemic treatments used for psoriasis. As we included already-treated patients for this purpose, most of them presented with few or no lesions at the time of the study, making it difficult to measure the previously mentioned variables using skin tissue.

## CONCLUSION

The most significant finding of this study is that the psoriasis group, comprising patients treated both systemically and topically, showed higher miR-205 levels than the control group. This suggests that psoriasis patients who have already received treatment present an important upregulation of miR-205, which leads to a decrease in systemic inflammation and, thus, an improvement in skin lesions. Further studies are needed to confirm our hypothesis in larger and more homogenic groups of patients evaluated before and after systemic treatments. Another important finding of this study is a possible correlation between the upregulation of miR-205 and the downregulation of TNF-α in treated patients with psoriasis. Understanding how miR-205 acts in psoriasis could be an important step toward further knowledge regarding the response of this type of non-coding RNA to modern treatments and possibly a gateway to more targeted genetic treatments in the future.

## Data Availability

Further data are available from the first author upon reasonable request.
